# Complications associated with ureterorenoscopy (URS) related to treatment of urolithiasis: the Clinical Research Office of Endourological Society URS Global study

**DOI:** 10.1007/s00345-016-1909-0

**Published:** 2016-08-04

**Authors:** B. K. Somani, G. Giusti, Y. Sun, P. J. Osther, M. Frank, M. De Sio, B. Turna, J. de la Rosette

**Affiliations:** 1 Department of Urology, University Hospital Southampton, Southampton, UK; 2 Department of Urology, IRCCS San Raffaele Scientific Institute, Ville Turro Division, Milan, Italy; 3 Department of Urology, Changhai Hospital, Shanghai, China; 4 Department of Urology, Lillebaelt Hospital, Fredericia, Denmark; 5 Department of Urology, City Hospital Number 40, Yekaterinburg, Russia; 6 Urology Unit, Second University of Naples, Naples, Italy; 7 Department of Urology, Ege University School of Medicine, Izmir, Turkey; 8 Department of Urology, AMC University Hospital, Meibergdreef 9, 1105 AZ Amsterdam Z-O, The Netherlands

**Keywords:** Ureterorenoscopy, Complications, Clavien–Dindo, Urolithiasis

## Abstract

**Introduction:**

Ureterorenoscopy (URS) is a popular and growing option for management of ureteric and renal stones. The CROES URS Global Study was set up to assess the outcomes of URS in a large worldwide cohort of patients involving multiple centres. In this paper, we analysed the database for intra-operative and post-operative complications associated with ureterorenoscopy.

**Methods:**

The CROES database was established via collaboration between 114 centres in 32 countries worldwide, and information on both intra-operative and post-operative complications was collected electronically between January 2010 and October 2012.

**Result:**

On analysis of a total of 11,885 patients, the overall complication and stone-free rates were found to be 7.4 and 85.6 %, respectively. The intra-operative and post-operative complication rates were 4.2 and 2.6 %, respectively, and in total 5 deaths were reported in the study period. Taking into account different world economies, there were no differences in the complication rates between the developing and developed nations or between different centres from different continents.

**Conclusion:**

Ureterorenoscopy is a safe and effective procedure for treatment of stones, the outcomes of which are broadly comparable in different parts of the world for similar patient and stone demographics.

## Introduction

Ureterorenoscopy (URS) is used for management of urolithiasis and is often preferred due to its higher stone-free rate (SFR) compared to shockwave lithotripsy (SWL), and lower complication rate compared to percutaneous nephrolithotomy (PNL) [1]. Most important complications of ureterorenoscopy are up-migration [1, 2] of stones, higher retreatment rates [1] due to incompletely removed stones (specifically in larger stones), and damage to the ureter [1–3] (from minor mucosal damage to avulsion) as a consequence of access sheath placement or difficulties with negotiating the ureter and rarely due to repetitive in and out movement of instruments.

Most recent guidelines [4] state that the overall complication rate after URS is 9–25 %. Most complications are minor and do not require intervention, and ureteral avulsion and strictures are rare (<1 %). There is no statement on classification of complications by the EAU. In the literature, several classification scoring systems can be found, such as the Clavien–Dindo [5, 6] classification system, the Satava [7] classification system, and the PULS [8] classification system. The Clavien–Dindo classification system is the most widely used scoring of complications in surgical procedures. However, both the Satava and PULS scoring systems are URS specific.

The Clinical Research Office of the Endourological Society (CROES) URS Global Study was set up to assess the indications for URS and its treatment outcomes. Important aspects of these outcomes were the nature and severity of complications. In this study, we describe the intra-operative and post-operative complications associated with URS treatment from the CROES database.

## Methods

The CROES URS Global Study consists of 11,885 patients at 114 centres in 32 countries. All these 114 centres used collected data electronically through an internet website (www.croesoffice.org), by which data were encrypted and stored in a central database at the CROES office. This database includes both intra- and post-operative complications which were collected between January 2010 and October 2012. Details on data collection have been previously described elsewhere [9].

The location of these stones was ureteric (*n* = 8676, 73 %) and renal (*n* = 1852, 15.6 %), and the majority of ureteric stones were in the distal ureter (42 %).

### Complications

During the procedure, a standardized case report form (CRF) was used to collect details of any complication, which included information on the relationship of this specific event with the type of stone treatment. To define intra-operative complications, all predefined categories (uneventful, bleeding, ureteral perforation, failed access, conversions, and avulsion) were used as dichotomous variables (either having, or not having that specific complication), which allowed more than one option at the same time. Complications that could not be registered using any of the predefined categories could be classified as ‘other’. The free text that was captured with the ‘other’ option was further categorized with data mining. This categorization was based on URS-related complications known from the literature and included stone migration (proximal or extraureteral), mucosal injury, inability to reach a stone, and malfunction of instruments [7]. Descriptions that did not match any of these categories were examined manually and were classified as suspicious of tumour, impacted stones, encrusted stent, infection related, complications not directly related to URS or undefined complications.

After the procedure, the standardized Clavien–Dindo scoring system for surgical procedures [5, 6] was used. Post-operative complications were classified using the predefined categories: bleeding, fever, urinary tract infection (UTI), pulmonary embolism, cerebro-vascular accident (CVA), sepsis, acute abdomen, or acute myocardial infarction (AMI). Again, the option ‘other’ was available, in which the possibility was given to describe the complication in free text. The free text was categorized with data mining into one of the predefined variables, or into an additional category: pain, urinary retention, stent misplacement, nausea and vomiting, respiratory, or allergic reaction. The advantage of using the Clavien–Dindo scoring system is that in addition to the nature of the complications, it also covers grades of severity of these complications.

### Statistical analyses

We calculated the actual numbers and percentages of complications. Analyses on consequences were performed with a simplified dichotomous variable described as ‘having’ or ‘not having’ complications and were subdivided into ‘intra- and post-operative complications’ ranging from ‘minor’ to ‘major’ complications.

All analyses were performed using STATA version 13, StataCorp LP, College Station, USA (www.stata.com).

## Results

Out of 11,885 patients, 874 (7.4 %) had a complication, with a stone-free rate (SFR) of 85.6 %. Table 1 shows descriptive information of patients with and without complications. For 18 (0.2 %) patients, no information was available regarding complications. In Table 2, the nature of these complications is listed.

**Table 1:** Descriptive information on the CROES URS Global study

	Patients with complications *N* = 874	Patients without complications *N* = 10,993
*Pre-operative characteristics*		
Age	51.7 (16.3)	48.4 (15.8)
Gender		
Male	556 (63.7)	7145 (65.0)
Female	317 (36.3)	3842 (35.0)
BMI	26.7 (5.2)	27.0 (5.4)
Comorbidity		
CVD (including DM)	357 (40.9)	3233 (29.4)
Prednisone	14 (1.6)	92 (0.9)
Crohn’s disease	8 (0.9)	51 (0.5)
ASA		
I	324 (39.3)	5672 (55.4)
II	356 (43.2)	3635 (35.5)
III	137 (16.6)	884 (8.6)
IV	7 (0.9)	56 (0.6)
Stone location		
Ureteral stones	209 (62.9)	8068 (73.4)
Renal stones	550 (23.9)	1597 (14.5)
Combined procedure	83 (9.5)	975 (8.9)
Previous stone treatment	456 (52.2)	4482 (40.8)
Congenital abnormalities		
Horse shoe	5 (0.6)	40 (0.4)
Malrotation	1 (0.1)	16 (0.2)
Ectopic	5 (0.6)	23 (0.2)
Solitary kidney	38 (4.4)	274 (2.5)
Pre-operative stent placement	194 (22.3)	1985 (18.1)
*Intra-operative characteristics*		
Operation time (min)	50 (33–75)	40 (25–60)
URS type		
Semi rigid	562 (64.5)	8204 (74.9)
Flexible	174 (20.0)	1607 (14.7)
Both	135 (15.5)	1138 (10.4)
Antibiotics	775 (89.3)	9019 (83.0)
Access*		
Balloon	72 (8.2)	1505 (13.7)
Access Sheath	252 (28.8)	2011 (18.3)
Guidewire	722 (82.6)	8475 (77.2)
Other	20 (2.3)	264 (2.4)
Fragmentation device		
US	25 (2.9)	121 (1.1)
Laser	413 (47.5)	5500 (50.2)
Pneumatic	180 (20.7)	3456 (31.5)
EHL	5 (0.6)	29 (0.3)
Other	4 (0.5)	210 (1.9)
None	242 (27.9)	1679 (15.3)
*Post-operative characteristics*		
Retreatment including readmission	407 (46.6)	1490 (13.6)
SFR (treated area)	514 (59.8)	9682 (89.9)
LOHS (days)	2 (1–4)	1 (1–2)
Post-operative stent placement	769 (88.8)	8888 (81.0)
*Hospital characteristics*		
Economy		
Developed region: G7	281 (32.2)	2828 (25.7)
Developed region: non-G7	286 (32.7)	2776(25.3)
Emerging region: BRIC	107 (12.2)	2102 (19.1)
Emerging region: MICT	100 (11.4)	1233 (11.2)
Emerging region: G20	92 (10.5)	1974 (18.0)
Developing region	8 (0.9)	81 (0.7)
Continent		
Africa	40 (4.6)	397 (3.6)
Asia	231 (26.4)	4478 (40.7)
Europe	495 (56.6)	4721 (42.9)
North America	65 (7.4)	776 (7.1)
Oceania	6 (0.7)	53 (0.5)
South America	37 (4.2)	569 (5.2)
Case volume	4.8 (4.2–5.3)	5.0 (4.4–5.9)

Data are n (%) of patients for whom data were available. Percentages exclude missing values from denominators

* More than one option allowed

**Table 2:** Descriptive information on intra- and post-operative complications

	Number	%
*Intra-operative complication*		
Bleeding	167	1.41
Perforation	124	1.05
Failed access	198	1.67
Conversions	19	0.16
Other	100	0.84
Mucosal injury	15	0.13
Tumour (incidental)	7	0.06
Migration	15	0.13
Impacted stone	4	0.03
Encrusted stent	7	0.06
Infection	8	0.07
Complication NOT by URS	8	0.07
Undefined	36	0.30
*Post-operative complication*		
Bleeding	54	0.45
Fever	204	1.72
UTI	113	0.95
Pulmonary embolism	2	0.02
CVA	1	0.01
Sepsis	36	0.30
Acute abdomen	5	0.04
AMI	1	0.01
Pain	39	0.33
Urinary retention	13	0.11
Stent misplacement	12	0.10
Nausea and vomiting	3	0.03
Respiratory	5	0.04
Allergy	5	0.04
Other	17	0.15

**Fig. 1:**
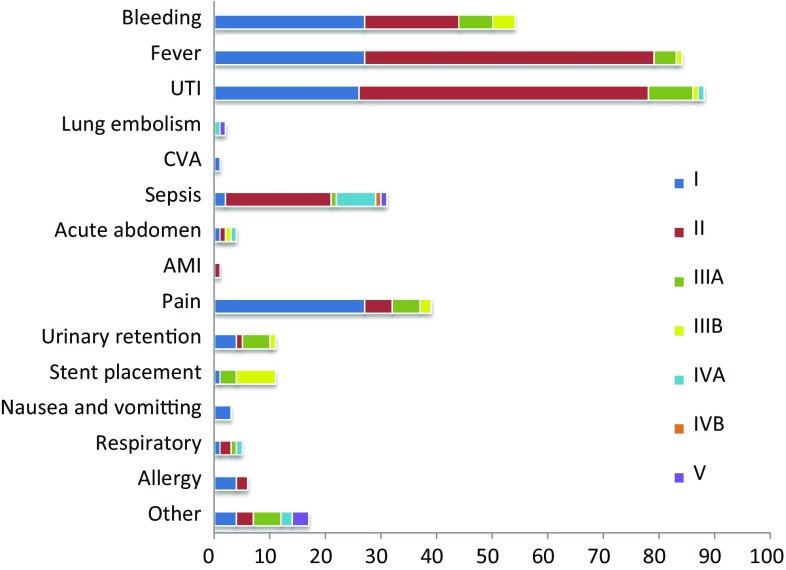
Clavien score by type of post-operative complication

Combining information on intra- and post-operative complications 55 (0.5 %) had both intra- and post-operative complications with 507 (4.2 %) and 311 (2.6 %) having isolated intra-operative and post-operative complications, respectively. Figure 1 shows the severity scores of all post-operative complications. Due to the nature of the intra-operative complication registration, the severity could not be elicited for the intra-operative complications. The vast majority of lower grades (I–II) of the Clavien classification scores were related to bleeding, fever, urinary tract infection (UTI), and pain. Events with Clavien grade III or IV were related to sepsis, stent misplacement, urinary retention, or complications from the ‘other’ category. The higher Clavien scores (IVA and IVB) that were scored post-operatively were scored in Australia, Chile, Czech Republic, Egypt, Israel, Japan, USA, and The Netherlands. There were no major differences found in severity of complications between continents (comparing Europe, Asia, North America, Africa, Oceania, and South America). Specifically, there were no differences in the higher Clavien scores. The only difference was that Europe, Oceania, and Africa had more grade 2 scores compared to Asia, North America, and South America, which had more grade 1 scores. Neither was there any difference in complications between different world economies (on comparing developed region G7, non-G7, BRIC, MIKT, G20 and the developing regions of the world).

As shown in Fig. 1, a total of 5 patients (0.04 %) died post-operatively during the 3-month follow-up period. Causes of death were described as fatal pulmonary embolism, sepsis, cardiac dysrhythmia, cardiac-related death and finally a case of acute bronchopneumonia and multi-organ failure. There were no intra-operative deaths reported from this worldwide series.

The pre- and post-operative stenting rates were 18.2 and 81 % for patients without complications, compared to 22.4 and 87 % for patients with complications. The median case volume per centre for patients without complications was higher (*n* = 155) compared to patients with complications (*n* = 120).

## Discussion

### Principle findings of the study

In this CROES study, the worldwide complications among 11,885 patients who underwent URS are presented. The most frequent complications were fever, failed procedures and bleeding, with a total of five post-operative deaths. The overall complication rate (7.4 %) was relatively low and acceptable for a mixed cohort of unselected patient population.

There were no differences in the overall rates of complications between different continents and economies (developed versus developing countries) suggesting that URS safety is comparable across the world.

### Classification of URS complications

One of the reasons for a low complication rate might be a slight selection bias for these patients who might be fitter compared to those receiving other forms of treatment. Another potential reason is the use of Clavien–Dindo classification system. Although the use of the Clavien–Dindo classification system for intra-operative complications is widely accepted in surgical disciplines, this may not be the best suitable scoring system for URS treatment. For this reason, the Satava classification system as proposed by Tepeler et al. [7] which is used in minimally invasive procedures might be better suited. This classification system describes complications that are URS specific, such as mucosal damage. Another proposed URS-specific classification system is the Post-Ureteroscopic Lesion Scale (PULS) [8]. In the present study, the intra-operative complications were classified with predefined categories covering topics of the Satava and PULS scoring system, and the post-operative complications were classified by the Clavien–Dindo system. As this study is a registry representing real-life situations, we chose to use the more general Clavien–Dindo classification system as it is used by majority of the centres worldwide.

### Meaning and weakness of the study

One may question if the present manuscript has added value within the perspective of previous (large) single-centre studies, systematic reviews, and meta-analyses. Since this is not only the largest URS database, but also a database representing, prospectively collected data in a structured database resulting in homogenous data collection, this study provides added value to our insights into global outcomes of URS procedures.

A major limitation of the study is the small number of complications. Consequently, comparison of outcomes of patients with and without complications cannot be supported by statistical tests for differences. In general, when small groups (rare case disease) are to be compared, techniques as used case–control analyses are more suitable [10]. Hence, no matter how large the database, the percentage of cases matters.

Another possible limitation is linked to the Clavien–Dindo classifications system, and we may have suffered underreporting in minor self-limiting complications as described by Ibrahim [2]. One of these underreported self-limiting complications may be mucosal damage, and there is some debate whether or not mucosal damage is simply part of the procedure, or a complication worth mentioning and grading [8, 11]. Reasons for procedure-related mucosal damage may be the repetitive in and out movement of instruments, but also inappropriate use of instruments (for example, basketing) can be debated. Another underreported complication may be haematuria [2], which is not well reported in our study. This could well be a natural self-limiting part of the procedure rather than a complication. Also the use of information from the ‘other’ category might have been underreported due to the way of collecting this information. Similarly, up-migration or retropulsion of stone may not be a complication in itself but increases the total operative time or potentially the need for further treatment of this stone at a later date.

In our study, we have used predefined categories and highly frequent mentioned events as categories of complications. However, the complications that do not happen very often, or that users may be unlikely to present as complications, like those described in the manufacturer and user facility device experience (MAUDE) database, such as locked deflection, which is a rare case technical complication may not be captured [12]. This may also be the case for emergency ureterorenoscopy [13], as this was only performed in some but not all reporting centres. Unfortunately, information on elective or emergency procedures was not captured in the CROES URS Global study.

### Areas of future research

To put URS-related complications into perspective, we can compare the outcomes with PNL or SWL. One of the advantages of SWL is the possibility to perform a procedure without anaesthesia and without any invasiveness into the human body [4]. Infection-related complications with URS may be reduced using disposable material (scopes, laser-tips, baskets); however, its invasive nature unequivocally is related to potential infectious complications. For the same reason, PNL has higher complication rates (CROES PCNL = 20.5 %) [14]. More understanding on higher complication rates for pre-stented patients is needed, as it might simply be a reflection of the complexity of the case rather than being related to the stent in itself.

For stone and patient demographics, apart from comparing outcomes with all treatment types, it would also be ideal to standardize and predefine the treatment decisions, which vary widely amongst different healthcare setups worldwide. Only when this is achieved, we can truly compare outcomes and define complications, which are truly accepted benchmark worldwide.

## Conclusions

Ureterorenoscopy is a safe and effective procedure for treatment of stones, the outcomes of which are broadly comparable in different parts of the world for similar patient and stone demographics.

## Appendix: Clavien classification of surgical complications

I. Any deviation from the normal post-operative course without the need for pharmacological treatment or surgical, endoscopic and radiological interventions.
II. Requiring pharmacological treatment with drugs other than such allowed for grade I complications.

*Blood transfusions and total parenteral nutrition are also included.

III. Requiring surgical, endoscopic, or radiological intervention
  IIIA. Intervention not under general anaesthesia
  IIIB. Intervention under general anaesthesia
IV. Life-threatening complication (including CNS complications)‡ requiring IC/ICU-management
  IVA. Single organ dysfunction (including dialysis)
  IVB. Multi organ dysfunction
V. Death of a patient

*D: If the patients suffer from a complication at the time of discharge, the suffix ‘d’ (for ‘disability’) is added to the respective grade of complication. This label indicates the need for a follow-up to fully evaluate the complication.

Modified Clavien–Dindo scoring system [5, 6].

## Abbreviations

AMI: Acute myocardial infarction
CRF: Case report form
CROES: Clinical Research Office of Endourological Society
CVA: Cerebro-vascular accident
CVD: Cardiovascular disease
DM: Diabetes mellitus
EHL: Electro-hydraulic lithotripsy
LOHS: Length of hospital stay
PNL: Percutaneous nephrolithotomy
SFR: Stone-free rate
SWL: Shockwave lithotripsy
URS: Ureterorenoscopy
US: Ultrasound
UTI: Urinary tract infection
